# Unmasking the large and highly repetitive genome of Phlox reveals a complex evolutionary history of speciation with gene flow

**DOI:** 10.64898/2026.09.01.748350

**Published:** 2026-09-04

**Authors:** Felix L Wu, Danielle E Khost, Patrick F McKenzie, Samridhi Chaturvedi, Grace A Burgin, Timothy B Sackton, Robin Hopkins

## Abstract

The large size and high repetitive content of many plant genomes have hindered elucidation of how the fundamental processes of evolution give rise to speciation. Here we present chromosome-level assemblies of 6 gigabase genomes for four closely related Phlox wildflower species. We generate extensive population genetic data for the three well-studied annual species P. drummondii, P. cuspidata, and P. roemeriana, including structural variation from whole-genome long-read resequencing data. The whole-genome assemblies reveal extensive differences in amount and distribution of genetic variation within and between species, reflective of differences in life-history strategies, mating systems, and edaphic specialization. The population genetic data exposes a history of widespread and consistent gene flow between all three annual Phlox species throughout their divergence and speciation. Our unmasking of structural variants and repetitive elements exposes rich and dynamic forms of genetic variation that show strong phylogenetic signal and pervasive patterns of gene flow. Our results lay a foundation for untangling the highly complex genomes of plants to make advancements in our understanding of the processes of speciation and divergence.

## Full Text Availability

The license terms selected by the author(s) for this preprint version do not permit archiving in PMC. The full text is available from the preprint server.

